# An interpretable predictive model for depression risk in diabetic patients: A web-based application using NHANES data

**DOI:** 10.1097/MD.0000000000049402

**Published:** 2026-06-26

**Authors:** Yishi Li, Tong Ren, Guanghong Zhou, Zhi Li, Chunyan Hu, Junfeng Zhao, Tianlin Guo, Yongqing Jiao, Chuanguang Zhou, Xun Wang

**Affiliations:** a Department of Neurointerventional Center, The Third People’s Hospital Affiliated Dalian University of Technology, Dalian, Liaoning, China; b Baotou Medical College, Inner Mongolia University of Science& Technology, Baotou, Inner Mongolia, China.

**Keywords:** depression, diabetes, machine learning, NHANES, predictive model

## Abstract

Depression is a common comorbidity in individuals with diabetes and is associated with adverse clinical outcomes. Early identification of high-risk individuals remains challenging due to the multifactorial and nonlinear nature of depression risk. Machine learning (ML) may enhance risk prediction but requires appropriate handling of class imbalance and sufficient interpretability for clinical application. The present study aimed to develop and rigorously evaluate an interpretable, class-imbalance-aware ML model for predicting depression risk among adults with diabetes, using nationally representative data from the National Health and Nutrition Examination Survey (NHANES). We analyzed cross-sectional data from 1140 adults with diabetes in the US (NHANES, 2007–2018). Depression was defined as a Patient Health Questionnaire-9 score ≥10. Predictors included demographic, clinical, lifestyle, and socioeconomic factors. Class imbalance was addressed using combined sampling and cost-sensitive learning. Seven ML models (logistic regression, support vector machine [SVM], random forest, adaptive boosting, decision tree, extreme gradient boosting, and categorical boosting [CatBoost]) were trained and evaluated. Model performance was assessed using the area under the receiver operating characteristic curve, with screening-oriented metrics optimized via threshold tuning. Model interpretability was examined using SHapley Additive exPlanations (SHAP), and clinical utility was evaluated using decision curve analysis. The SVM model demonstrated the most balanced performance after threshold optimization, achieving superior sensitivity and positive-class F1-score for depression detection. Key predictors identified by SHAP included chest pain, poverty-income ratio, sleep duration, sex, body mass index, physical activity, triglyceride levels, and diet quality (Healthy Eating Index–2020). Decision curve analysis indicated favorable net benefit for screening, particularly at lower risk thresholds. An interactive web-based application was developed to provide individualized risk predictions and explanations. An interpretable, imbalance-aware SVM model effectively predicts depression risk among adults with diabetes and supports individualized risk stratification, offering a potential tool for precision screening and early intervention.

## 1. Introduction

Depression is one of the most common psychiatric comorbidities among individuals with diabetes, with prevalence estimates generally ranging from 10% to 15%: approximately twice that observed in nondiabetic populations, and has been consistently associated with poorer glycemic control, reduced treatment adherence, increased complication rates, and higher all-cause mortality.^[1]^ Given the rising global prevalence of diabetes, which currently affects over 537 million people worldwide, and the substantial burden of depression, which impacts more than 350 million individuals globally and accounts for over 56 million disability-adjusted life years lost, early identification of individuals at high-risk of depression has become a critical public health and clinical priority.^[2–5]^

The relationship between diabetes and depression is complex and multifactorial, involving biological, behavioral, and psychosocial pathways. Metabolic dysregulation, chronic inflammation, HPA axis disturbances, sleep disturbances, lifestyle behaviors, and socioeconomic stressors have all been implicated in the development of depressive symptoms among patients with diabetes.^[6–8]^ Depression in this population not only heightens emotional distress but also complicates self-management, disrupts glycemic control, and elevates the risk of long-term complications. However, traditional screening approaches and conventional regression-based risk models typically rely on a limited number of predictors and assume linear associations, which may fail to capture the heterogeneous and nonlinear nature of depression risk in this population.

Recent advances in artificial intelligence (AI) and machine learning (ML) have enabled the development of predictive models that integrate multidimensional data to identify individuals at increased risk of depression. Previous studies have applied logistic regression (LR)-based clinical prediction models, passive sensing approaches, and deep learning methods using electronic health records to predict depression among patients with diabetes.^[8–10]^ While these efforts demonstrated the feasibility of AI-based prediction, several methodological limitations remain. Many studies relied on relatively small or highly selected samples, applied complex models without sufficient consideration of class imbalance, or focused primarily on predictive performance without addressing interpretability and individualized risk assessment. Moreover, highly complex models may be difficult to translate into routine clinical practice, particularly when sample sizes are limited and outcome prevalence is low.

Importantly, few existing studies have systematically incorporated a broad range of biological, behavioral, and psychosocial predictors, such as demographic factors, clinical indicators, laboratory results, health behaviors (e.g., physical activity (PA), sleep, diet quality), and socioeconomic determinants, while simultaneously addressing class imbalance and providing transparent, patient-level explanations of model predictions. This gap limits the clinical applicability of current AI-based approaches and constrains their alignment with the principles of precision psychiatry.

The present study aimed to develop and compare multiple ML models for the prediction of depression among adults with diabetes using nationally representative NHANES data. We systematically integrated demographic characteristics, clinical indicators, laboratory parameters, lifestyle behaviors, dietary quality measures, and socioeconomic determinants as candidate predictors. To address class imbalance, we combined resampling strategies with cost-sensitive learning and optimized screening-oriented performance through threshold tuning. Model discrimination was evaluated using area under the ROC curve (AUC) and related metrics; clinical utility was assessed via decision curve analysis (DCA), and interpretability was ensured using SHapley Additive exPlanations (SHAP) to provide transparent, individualized risk explanations. Through this framework, we sought to construct a clinically applicable, interpretable, and precision-oriented screening tool for depression in individuals with diabetes.

## 2. Materials & methods

### 2.1. Study design and population

This study analyzed data from the National Health and Nutrition Examination Survey (NHANES) collected between 2007 and 2018. The NHANES is a nationally representative, cross-sectional survey of the noninstitutionalized U.S. population, combining household interviews with standardized physical examinations to assess health and nutritional status. Of 59,842 participants across 6 survey cycles (2007–2018), we included adults aged ≥18 years who self-reported a physician diagnosis of diabetes. We excluded individuals with a history of gestational diabetes only, missing data on depressive symptoms measured by the Patient Health Questionnaire-9 (PHQ-9), or incomplete information for prespecified covariates (demographic, lifestyle, clinical, or laboratory variables). After exclusion, 1140 participants with diabetes were included in the analysis.

### 2.2. Diabetes identification

To reduce diagnostic misclassification, the diabetes status was determined using self-reported questionnaire data and laboratory measurements of glycohemoglobin. Participants were classified as diabetic if they reported ever being told by a healthcare professional that they had diabetes and had an glycohemoglobin level of ≥6.5%.^[11]^ Patients with a history of gestational diabetes only were excluded. Individuals with missing PHQ-9 data for depressive symptom assessment or with incomplete information on prespecified covariates were also excluded.

### 2.3. Depression assessment

Depression was assessed using the PHQ-9, which measures depressive symptoms experienced during the previous 2 weeks. A 2019 individual participant data meta-analysis by Levis et al (*n* = 17,357) reported a sensitivity of 0.88 (95% CI: 0.83–0.92) and a specificity of 0.85 (95% CI: 0.82–0.88) for the commonly used cutoff score.^[12]^ The PHQ-9 consists of 9 items, each rated from 0 to 3 (0 = “not at all,” 1 = “several days,” 2 = “more than half the days,” 3 = “nearly every day”), with total scores ranging from 0 to 27. In this study, a cutoff of ≥10 was used to define depression, whereas scores < 10 indicated no depression.^[13]^

### 2.4. Predictors

On the basis of previous studies,^[8–10]^ we included a range of demographic, lifestyle, clinical, and laboratory predictors. Demographic variables included sex (male or female), age, race/ethnicity (Mexican American, other Hispanic, non-Hispanic White, non-Hispanic Black, other/multiracial), educational level (less than high school, high school or equivalent, some college or above), marital status (married/living with partner, widowed/divorced/separated, never married), and poverty-income ratio (PIR). Physical examination data included body mass index (BMI) calculated as weight (kg) divided by height (m^2^). Lifestyle factors included smoking (defined as lifetime consumption of ≥100 cigarettes), alcohol use (≥12 drinks in any year), and participation in moderate-intensity leisure activities. Medical history included hypertension, diabetes, and chest pain. The sleep duration was self-reported. Laboratory tests included total cholesterol (mol/L) and (High-density lipoprotein cholesterol; mmol/L).

Dietary quality was assessed using the healthy eating index–2020 (HEI-2020), which evaluates adherence to the 2015 Dietary Guidelines for Americans. The index consists of 13 components: 9 adequacy components (total fruits, whole fruits, total vegetables, greens and beans, whole grains, dairy, total protein foods, seafood and plant proteins, and fatty acids) and 4 moderation components (refined grains, sodium, added sugars, and saturated fats). The overall score ranged from 0 to 100, with higher scores reflecting better diet quality. Participants with an average HEI-2020 score of ≥60 across 2 days were classified as meeting the dietary recommendations.^[14]^ The R package^[15,16]^ was used for calculation, yielding results consistent with the official NHANES data.

### 2.5. Data preprocessing

To ensure analytic accuracy and reliability, individuals with missing data were excluded rather than applying imputation, thereby minimizing potential bias. The final dataset was randomly divided into training (80%) and testing (20%) subsets.

Several baseline characteristics were recorded as categorical variables. Alcohol consumption, hypertension, smoking status, PA, and chest pain were dichotomized as “Yes” or “No.” Sex was coded as “Male” or “Female.” Race/ethnicity was categorized into 5 groups: Mexican American, other Hispanics, non-Hispanic White, non-Hispanic Black, and other/multiracial. Education was classified into 5 levels: <9th grade, 9th–11th grade (including 12th grade without diploma), high school graduate or general educational development equivalent, some college or associate’s degree, and college graduate or higher. Marital status was grouped into 6 categories: married, widowed, divorced, separated, never married, and living with a partner. These categorizations were consistently applied across descriptive analyses and regression models.

### 2.6. Handling of class imbalance

Before implementing advanced modeling strategies, an exploratory model evaluation was first conducted using conventional classifiers with default probability thresholds. This preliminary analysis revealed a marked outcome imbalance: the prevalence of depression was substantially lower than that of non-depression, with a negative-to-positive ratio of 6.41:1. Under these conditions, several models demonstrated apparently high overall accuracy but poor sensitivity for detecting depressive cases, indicating a tendency to bias predictions toward the majority (non-depressed) class.

To address this issue and improve minority-class recognition, multiple complementary imbalance handling strategies were applied. First, class distributions were explicitly assessed in the training dataset to guide subsequent modeling choices. To enhance minority-class representation during training, combined over- and under-sampling was performed using the Synthetic Minority Oversampling Technique + Edited Nearest Neighbours algorithm, which synthesizes new minority samples while simultaneously reducing noisy majority observations.^[17,18]^

In parallel, all classification models incorporated cost-sensitive learning to penalize misclassification of depressive cases more heavily. LR, SVM, and decision tree models were trained using balanced class weights. Ensemble models applied algorithm-specific weighting approaches, including class_weight=“balanced_subsample” for RandomForest, scale_pos_weight for extreme gradient boosting (XGBoost), and auto_class_weights=“Balanced” for CatBoost. These strategies were intended to reduce bias toward the majority class and improve discrimination for participants with depression.

### 2.7. ML models

Seven supervised ML algorithms were evaluated, including LR, SVM, RandomForest, Adaptive Boosting, DecisionTree, XGBoost, and CatBoost. LR was included as a baseline linear model due to its interpretability and widespread use in clinical research. SVM was selected to capture potential nonlinear decision boundaries while maintaining robustness in high-dimensional and imbalanced settings.

Tree-based ensemble models (RandomForest, XGBoost, and CatBoost) were included to account for complex nonlinear interactions and feature hierarchies. All models were trained using 5-fold stratified cross-validation, and hyperparameters were optimized via randomized search with the AUC as the optimization objective.

### 2.8. Threshold optimization and model evaluation

Given the pronounced class imbalance and the screening-oriented nature of this study, model performance was not evaluated using a fixed probability threshold of 0.5. Instead, optimal decision thresholds were determined for each model based on the precision-recall curve by maximizing the F1 score for the positive class.

Model discrimination was assessed using the AUC. Additional performance metrics included accuracy, macro-averaged F1 score, positive-class F1 score, sensitivity (recall), precision, and confusion matrix–derived measures (true positives, false positives, true negatives, and false negatives). All metrics were calculated using the optimized probability thresholds.

This evaluation strategy was designed to prioritize the detection of depression cases while controlling false-negative errors, consistent with the objectives of clinical screening.

All ML models were developed and validated using Python (version 3.12), and statistical analyses were performed using R (version 4.4.2). The median and interquartile range were reported for continuous variables that did not follow a normal distribution, and the Mann–Whitney *U* test was used for group comparisons. Categorical variables are summarized as frequencies and percentages, and differences between groups were assessed using the chi-square test.

### 2.9. Decision curve analysis

**Figure 1.**
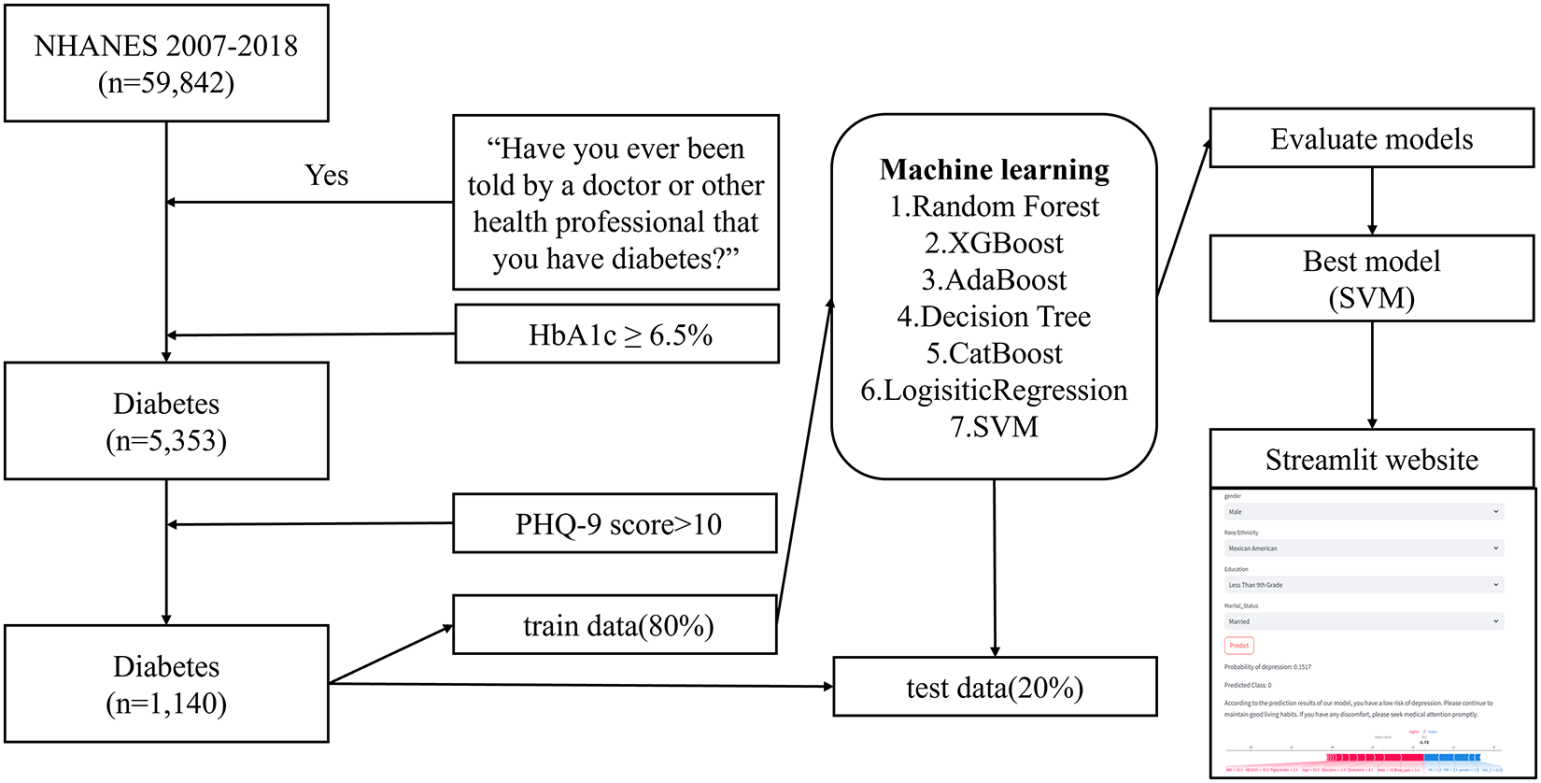
Overall analytical framework of the study. This figure illustrates the complete workflow for developing and validating a machine learning-based depression risk prediction model among adults with diabetes using NHANES data (2007–2018). After participant selection and outcome definition, multidimensional predictors encompassing demographic, socioeconomic, clinical, lifestyle, laboratory, and dietary factors were extracted. Class imbalance was explicitly addressed through a combination of resampling techniques and cost-sensitive learning. Multiple machine learning models were trained, optimized, and evaluated. The final model was interpreted using SHapley Additive exPlanations (SHAP), its clinical utility was assessed via decision curve analysis (DCA), and an interactive web-based application was developed to enable individualized risk prediction and transparent interpretation. SVM = support vector machine.

DCA was performed to evaluate the clinical utility of each model by quantifying the net benefit across a range of threshold probabilities.^[19,20]^ DCA incorporates the relative harms of false positives and false negatives and provides insight into the potential value of a predictive model in real-world clinical decision-making.^[21]^ Models with higher net benefit across clinically relevant threshold ranges were considered to have greater applicability for depression screening among patients with diabetes. Figure 1 summarizes the overall analytical framework of the study, illustrating the complete workflow from participant selection and feature extraction to imbalance-aware model development, interpretability analysis, clinical utility evaluation, and deployment of an interactive prediction tool.

## 3. Results

### 3.1. Baseline characteristics of participants

A total of 1140 participants were included in the study, with 912 and 228 participants assigned to the training and testing sets, respectively (Table 1). The overall cohort had a median age of 61 years and consisted of 588 males (51.6%) and 552 females (48.4%), among whom 154 were diagnosed with depression. In the training set, the median age was 61 years, comprising 460 males (50.4%) and 452 females (49.6%), with 127 cases of depression. The median age of the testing set was 60 years, with 128 males (56.1%) and 100 females (43.9%), including 27 individuals with depression. No significant differences we/re observed between the training and testing sets across all baseline variables (*P* > .05).

**Table 1:** Baseline characteristics of participants.

Variable		Category	Overall (n = 1140)	Train (n-912)	Test (n = 228)	*p*-value
**Alcohol**		Yes	700 (61.40%)	561 (61.51%)	139 (61.30%)	0.9
		No	440 (38.60%)	351 (38.49%)	89 (38.70%)	
**Hypertension**		Yes	822 (72.11%)	654 (71.71%)	168 (73.70%)	0.6
		No	318 (27.89%)	258 (28.29%)	60 (26.30%)	
**Gender**		Male	588 (51.58%)	460 (50.44%)	128 (56.10%)	0.1
		Female	552 (48.42%)	452 (49.56%)	100 (43.90%)	
**Race/Ethnicity**		Mexican American	189 (16.58%)	153 (16.78%)	36 (15.79%)	0.7
		Other Hispanic	132 (11.58%)	101 (11.07%)	31 (13.60%)	
		Non-Hispanic White	455 (39.91%)	371 (40.68%)	84 (36.84%)	
		Non-Hispanic Black	278 (24.39%)	221 (24.23%)	57 (25.00%)	
		Other race - including multiracial	86 (7.54%)	66 (7.24%)	20 (8.77%)	
**Education**		> 9th grade	154 (13.51%)	131 (14.36%)	23 (10.10%)	0.2
		9–11th grade (includes 12th grade with no diploma)	204 (17.89%)	153 (16.78%)	51 (22.40%)	
		High school Grad/GED or equivalent	280 (24.56%)	223 (24.45%)	57 (25.00%)	
		Some college or AA degree	302 (26.49%)	242 (26.54%)	60 (26.30%)	
		College graduate or above	200 (17.54%)	163 (17.87%)	37 (16.20%)	
**Marital status**		Married	660 (57.89%)	520 (57.02%)	140 (61.40%)	0.2
		Widowed	153 (13.42%)	126 (13.82%)	27 (11.84%)	
		Divorced	165 (14.47%)	129 (14.14%)	36 (15.79%)	
		Separated	42 (3.68%)	34 (3.73%)	8 (3.51%)	
		Never married	86 (7.54%)	77 (8.44%)	9 (3.95%)	
	Living with partner		34 (2.98%)	26 (2.85%)	8 (3.51%)	
**Smoke**	Yes		567 (49.74%)	444 (48.68%)	123 (54.00%)	0.2
	No		573 (50.26%)	468 (51.32%)	105 (46.00%)	
**PA**	Yes		355 (31.14%)	288 (31.58%)	67 (29.40%)	0.6
	No		785 (68.86%)	624 (68.42%)	161 (70.60%)	
**Chest**_**pain**	Yes		377 (33.07%)	299 (32.79%)	78 (34.20%)	0.7
	No		763 (66.93%)	613 (67.21%)	150 (65.80%)	
**BMI**			31.3 (27.5–36.0)	31.2 (27.5–36.0)	31.3 (27.5–36.0)	1
**Age**			61.0 (48.0–71.0)	61.0 (47.0–71.0)	60.0 (49.8–70.0)	0.8
**Sleep**			7.0 (6.0–8.0)	7.0 (6.0–8.0)	7.0 (6.0–8.0)	0.8
**HEI2020**			51.6 (44.1–59.8)	51.6 (43.8–59.9)	52.3 (44.7–59.5)	0.9
**PIR**			1.75 (1.02–3.07)	1.73 (1.03–3.11)	1.8 (0.978–2.83)	0.8
**HDL_C**			46.0 (40.0–56.0)	46.0 (40.0–56.2)	45.0 (39.0–56.0)	0.4
**Triglycerides**			1.41 (1.01–2.04)	1.41 (1.02–2.04)	1.44 (1.0–2.04)	0.9
**Cholesterol**			4.47 (3.83–5.28)	4.47 (3.85–5.25)	4.42 (3.66–5.31)	0.6

Alcohol = defined as at least 12 drinks in any year, BMI = body mass index, HEI2020_ALL = healthy eating index (2020), PA = physical activity, PIR = poverty-income ratio, smoking = defined as lifetime consumption of at least 100 cigarettes.

### 3.2. Comparison of model performance

The performance of all ML models was evaluated on the independent testing dataset using optimized probability thresholds. While several models demonstrated comparable discrimination ability, the SVM achieved the most balanced and clinically relevant performance across multiple metrics.

SVM exhibited a high AUC alongside superior positive-class F1 score and recall after threshold optimization, indicating improved sensitivity for detecting depression cases. Although tree-based ensemble models such as CatBoost and XGBoost achieved competitive AUC values, their performance gains did not consistently translate into improved positive-class classification after threshold adjustment. The detailed performance metrics of all models are summarized (Table 2)

**Table 2:** Performance metrics of machine learning models on the independent testing set.

Model	AUC_ROC	AUC_PR	Accuracy	Balanced accuracy	F1 (macro)	F1 (+)	Recall (+)	Precision (+)
CatBoost	0.7837	0.2787	0.8509	0.4924	0.4597	0	0	0
RandomForest	0.783	0.3096	0.8509	0.4924	0.4597	0	0	0
SVM	0.7714	0.3878	0.6623	0.6415	0.5523	0.3304	0.6129	0.2262
XGBoost	0.7694	0.2799	0.8553	0.4949	0.461	0	0	0
Logistic	0.7665	0.3803	0.6798	0.6516	0.5654	0.3423	0.6129	0.2375
AdaBoost	0.7355	0.2567	0.864	0.5	0.4635	0	0	0
DecisionTree	0.607	0.1809	0.8202	0.5018	0.4946	0.0889	0.0645	0.1429

The table summarizes the evaluation of 7 machine learning algorithms for predicting depression among adults with diabetes, including CatBoost, RandomForest, SVM, XGBoost, LR, AdaBoost, and DecisionTree. Model performance was assessed using discrimination metrics (area under the receiver operating characteristic curve [AUC_ROC] and area under the precision-recall curve [AUC_PR]), overall accuracy, balanced accuracy, macro-averaged F1 score (F1 Macro), positive-class F1 score (F1(+)), recall (sensitivity) for the positive-class (Recall(+)), and precision for the positive-class (Precision(+)).

Given the screening-oriented objective of this study, SVM demonstrated a more favorable trade-off between sensitivity and precision, with fewer false-negative cases compared with ensemble-based models.

### 3.3. Rationale for selecting the final model

Although CatBoost and XGBoost demonstrated strong discrimination performance, SVM was selected as the final predictive model based on its superior performance in clinically relevant metrics and overall stability. After optimizing probability thresholds, SVM consistently achieved higher positive-class F1 scores and recall, which are critical for minimizing missed depression cases in screening settings.

In addition, SVM showed reduced sensitivity to class imbalance when combined with balanced class weights and threshold optimization, resulting in more stable confusion matrix profiles across validation folds. Compared with complex ensemble models, SVM also offers improved generalizability and reduced risk of overfitting in moderately sized epidemiological datasets.

**Figure 2.**
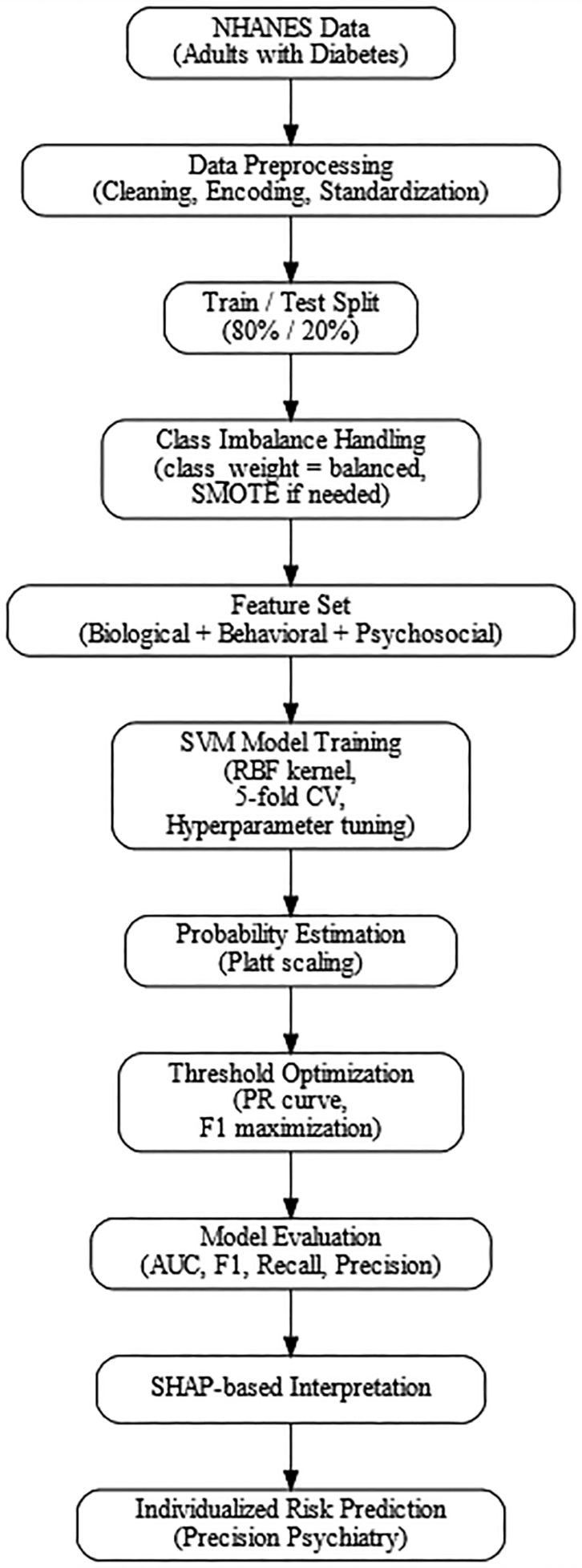
Workflow of the support vector machine–based depression risk prediction model. NHANES data were preprocessed and split into training and testing sets. Class imbalance was addressed using cost-sensitive learning and resampling. An SVM with an RBF kernel was trained using cross-validation and hyperparameter tuning. Probability calibration, threshold optimization, and SHAP-based interpretation enabled individualized depression risk prediction within a precision psychiatry framework. AUC = area under the ROC Curve, NHANES = National Health and Nutrition Examination Survey, PR = precision-recall, RBF = Radial Basis Function, SHAP = SHapley Additive exPlanations.

Taken together, these considerations support the selection of SVM as the final model for downstream interpretation and clinical applicability analyses. The overall modeling pipeline and architecture of the SVM-based framework are illustrated in Figure 2.

### 3.4. Results visualization

**Figure 3.**
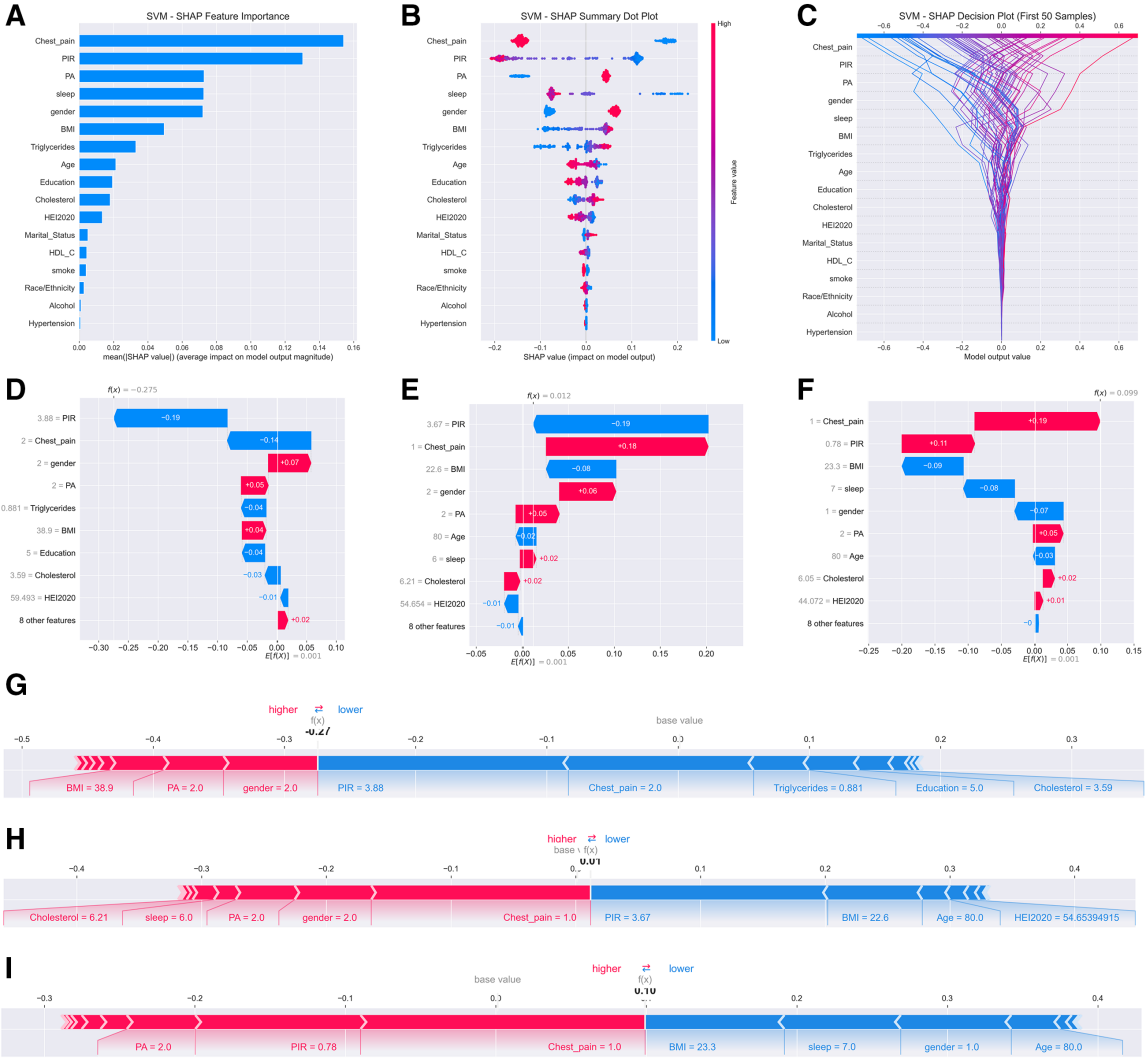
SHAP-based interpretability analysis of the final support vector machine (SVM) model. (A) Global feature importance ranking based on mean absolute SHAP values. (B) SHAP summary dot plot demonstrating both the magnitude and direction of each feature’s contribution across individuals. (C) SVM decision plot illustrating cumulative feature contributions along the decision boundary. (D–F) SHAP waterfall plots for 3 representative individuals, showing how predictors incrementally increase or decrease depression risk. (G–I) Corresponding SHAP force plots for the same individuals, visualizing the balance between risk-enhancing and protective factors at the individual level.

To enhance the interpretability of the final predictive model, SHapley Additive exPlanations (SHAP) were applied to the optimized SVM classifier.^[22]^ Global feature importance based on mean absolute SHAP values (Fig. 3A) identified chest pain, PIR, sleep duration, sex, BMI, PA, triglyceride levels, HEI-2020, and High-Density Lipoprotein Cholesterol as the most influential predictors of depression risk.

The SHAP summary dot plot (Fig. 3B) illustrates both the direction and magnitude of each feature’s contribution across individuals, revealing pronounced heterogeneity and nonlinear effects. The SVM decision plot (Fig. 3C) further demonstrates how multiple predictors jointly contribute to model decisions along the classification boundary.

Individual level interpretability is illustrated in Figures 3D–I, where SHAP waterfall and force plots depict how specific predictors increase or attenuate depression risk in representative individuals with and without depression. These visualizations provide transparent, patient-specific explanations for model predictions.

**Figure 4.**
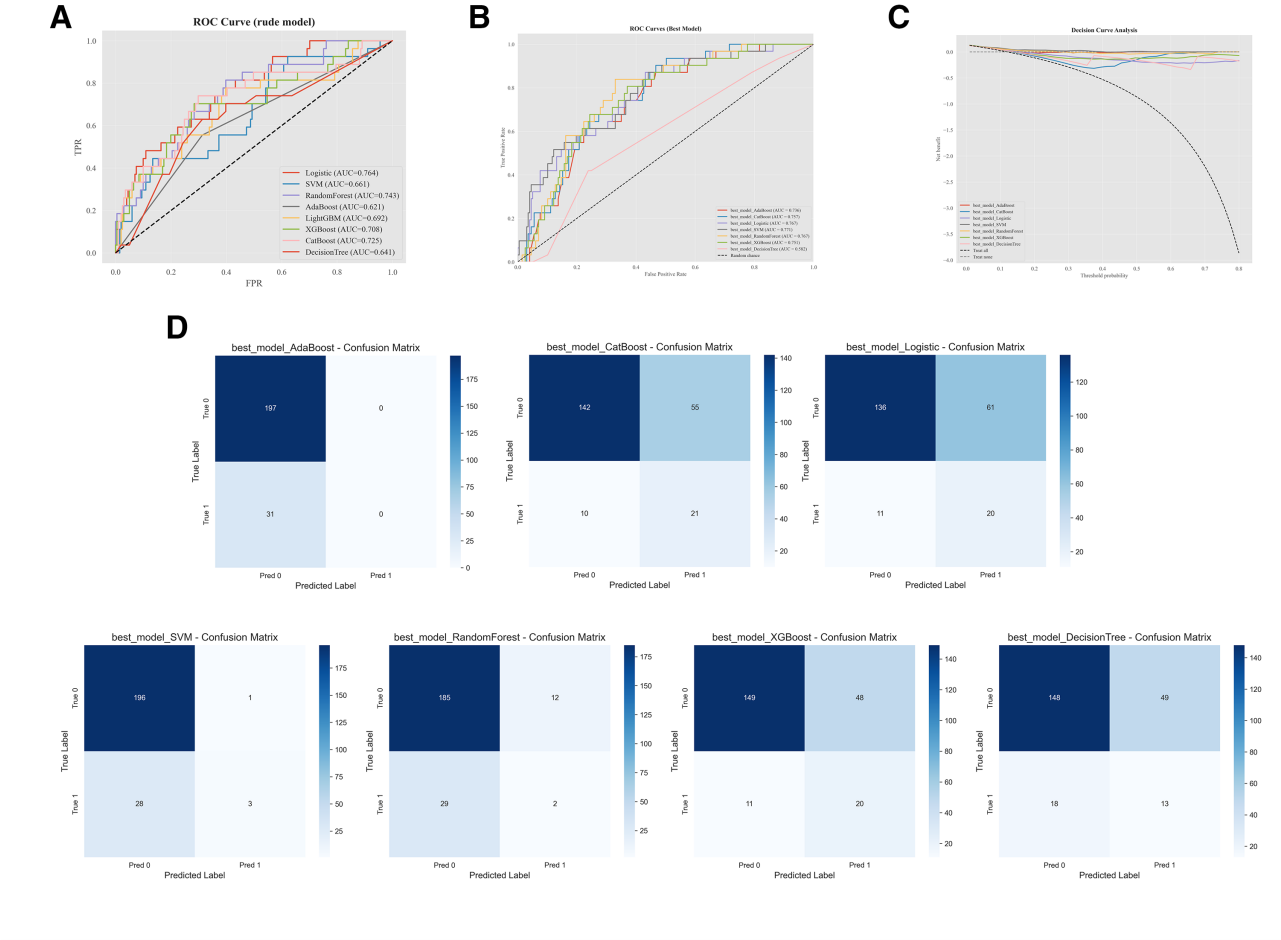
Comparison of model performance and clinical utility. (A) Performance of unadjusted models trained without explicit handling of class imbalance, demonstrating limited sensitivity for depression detection. (B) Performance of the optimized SVM model after combined resampling, cost-sensitive learning, and threshold optimization. (C) Decision curve analysis (DCA) of the final SVM model across a range of threshold probabilities compared with treat-all and treat-none strategies. (D) Confusion matrices of all evaluated models using optimized thresholds.

Model performance comparisons are presented in Figure 4. Unadjusted models (Fig. 4A) exhibited limited sensitivity for detecting depression despite acceptable discrimination. In contrast, the optimized SVM model (Fig. 4B) achieved a more favorable balance between sensitivity and precision. DCA (Fig. 4C) demonstrated a higher net benefit for the SVM model across clinically relevant low threshold probabilities. Confusion matrices (Fig. 4D) further highlighted the superior balance achieved by the SVM model in identifying depression cases.

**Figure 5.**
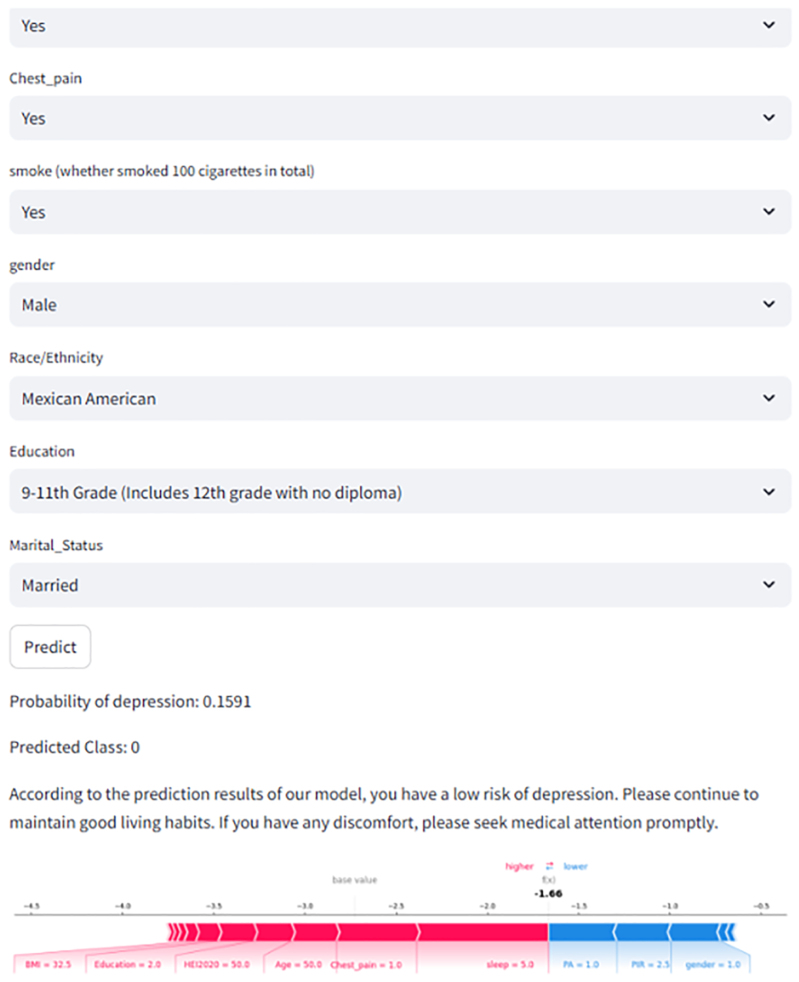
Web-based individualized risk prediction application. The application allows users to input individual level characteristics to generate personalized depression risk estimates among adults with diabetes. SHAP-based explanations, including force plots, are provided to visualize predictor contributions and support transparent risk communication.

Finally, Figure 5 presents the web-based prediction application, which integrates individualized risk estimation with SHAP-based explanations to support user-friendly and transparent implementation.

We deployed a web-based prediction model using the Streamlit framework. This webpage can predict patients’ depression risk and generate individualized SHAP force plots. The link is as follows: https://predict-model-9fwi94jmdoqqglh8sacw93.streamlit.app/

Figure 5. Screenshot of the web-based individualized depression risk prediction application.

## 4. Discussion

In this study, we developed and evaluated multiple ML models to predict depression risk among adults with diabetes using nationally representative survey data. Among all candidate models, the SVM demonstrated the most favorable overall performance after accounting for class imbalance and optimizing decision thresholds. Notably, the SVM achieved the highest AUC and the best balance between sensitivity, precision, and F1 score for the positive-class, indicating its robustness in identifying individuals at risk for depression in a low-prevalence setting.

A recent NHANES-based study by Tang et al similarly applied explainable ML approaches to predict depression risk in adults with type 2 diabetes mellitus using SHAP analysis.^[23]^ Their findings demonstrated that ML models, particularly XGBoost, can achieve strong overall discriminative performance in identifying depressive symptoms within diabetic populations. The present study extends this line of research by specifically focusing on class imbalance-aware prediction and clinically oriented screening optimization. Rather than prioritizing overall classification accuracy alone, our framework emphasized sensitivity, positive-class F1-score, and threshold-adjusted detection performance to improve identification of high-risk individuals in low-prevalence settings.

Using SHAP-based interpretability analyses, we identified several groups of predictors that consistently contributed to depression risk prediction. These predictors can be broadly categorized into metabolic burden, behavioral and lifestyle factors, psychosocial stressors, and socioeconomic indicators. Markers of physical health and metabolic burden, including body mass index, lipid profiles, and chest pain, were among the most influential features. These findings are consistent with prior evidence linking cardiometabolic dysregulation and chronic somatic symptoms to depressive symptomatology, potentially through shared inflammatory and neuroendocrine pathways. Notably, network and metabolomics studies reveal central connections between specific somatic symptoms of depression, such as low energy, hypersomnia, and weight increase, and a distinct lipid profile characterized by larger very-low-density Lipoprotein particle size, elevated triglycerides, higher levels of saturated fatty acids, and reduced HDL, collectively forming a cardiometabolic-like signature of depression.^[24,25]^ Furthermore, multilayer cardiovascular–depression network analyses highlight BMI and lipid measures as highly interconnected nodes, bridging both depressive symptoms and cardiovascular indicators.^[26]^ Sleep duration and PA have emerged as strong behavioral predictors of depression among individuals with diabetes, a relationship underscored by observed behavioral clustering in this population. For instance, in U.S. adults with diabetes, both sleep deprivation (<5 hours) and insufficient PA are more prevalent in those with diabetic retinopathy: a subgroup that also demonstrates higher depression prevalence, suggesting a co-occurrence of short sleep, inactivity, and depressive symptoms.^[27–29]^ Some studies further highlight that disrupted sleep, whether insufficient or, in the case of atypical depression, excessively extended, can disturb circadian regulation, worsen insulin resistance, and intensify depressive symptoms in diabetes.^[30,31]^ Together, these findings underscore the role of circadian disruption and sedentary behavior in elevating depression risk in diabetic patients, pointing to sleep and activity patterns as modifiable behavioral targets. Importantly, many of these predictors represent noninvasive and clinically accessible features, supporting the feasibility of implementing ML–assisted depression screening in routine diabetes management settings.

Socioeconomic and psychosocial variables, particularly the PIR and sex, also played a substantial role in model predictions. Lower socioeconomic status may exacerbate psychological stress, limit access to healthcare resources, and increase vulnerability to both metabolic and mental health disorders. Sex-specific differences in depression risk are well documented and may reflect a combination of biological susceptibility and gender-related psychosocial factors.^[32–34]^ Collectively, the importance of these features highlights the multifactorial nature of depression risk and supports the need for integrative predictive approaches.

Beyond overall predictive performance, a key strength of the proposed framework lies in its ability to generate individualized risk estimates. This distinction is clinically important because depression screening tools should prioritize minimizing missed high-risk cases rather than maximizing specificity alone, particularly in population-level preventive settings. By leveraging SHAP-based explanations, the model provides patient-specific insights into which factors increase or decrease predicted depression risk. This individualized interpretability aligns with the principles of precision psychiatry, facilitating risk stratification and enabling clinicians to tailor screening and preventive strategies based on each patient’s unique risk profile rather than relying solely on population-level thresholds. To bridge the gap between research and practice, we operationalized this model into a user-friendly, web-based application using the Streamlit framework. This deployment enhances the portability and accessibility of the tool, allowing for potential point-of-care use by clinicians or self-assessment by patients, thereby directly supporting the goal of early identification and personalized intervention.

Although ensemble tree-based models such as CatBoost demonstrated competitive performance, the SVM was ultimately selected because its threshold-adjusted predictions achieved a more favorable balance between sensitivity and positive predictive performance under class-imbalanced conditions. This screening-oriented optimization strategy may be particularly important in depression detection tasks, where false-negative predictions could delay recognition and intervention in vulnerable individuals. In addition, the SVM maintained stable generalization performance despite the relatively modest positive sample size and facilitated efficient deployment within the web-based application framework.

Several limitations warrant consideration. Despite the use of a nationally representative dataset, the absolute number of participants with depression was relatively small, reflecting the low-prevalence of the outcome. This imbalance may limit the stability of complex models and underscore the importance of cautious interpretation. Additionally, external validation was not performed, and future studies using independent cohorts are needed to confirm generalizability. The cross-sectional design further precludes causal inference. Although our study emphasized imbalance-aware screening optimization and individualized interpretability, prospective comparisons between different ML deployment strategies across diabetic subpopulations remain necessary. Nonetheless, we mitigated these concerns by applying class-weighted learning, cross-validation, threshold optimization based on precision-recall trade-offs, and transparent model interpretation. It should also be noted that the deployed application is a proof-of-concept tool; its clinical utility and impact require prospective evaluation in real-world settings.

## 5. Conclusions

In conclusion, this study demonstrates that an interpretable, imbalance-aware SVM framework can effectively support depression risk screening among adults with diabetes while providing individualized risk explanations. Complementing previous explainable ML studies in diabetic populations, our findings further highlight the importance of class imbalance handling, threshold optimization, and screening-oriented evaluation in improving the detection of high-risk individuals. The deployment of this model as an interactive web application represents a practical step toward clinically accessible precision screening and early intervention strategies for depression in metabolic populations.

## Author contributions

**Conceptualization:** Yishi Li, Tong Ren, Xun Wang.

**Funding acquisition:** Yishi Li, Tong Ren, Chuanguang Zhou, Xun Wang.

**Methodology:** Yishi Li, Guanghong Zhou, Zhi Li, Yongqing Jiao.

**Investigation:** Tong Ren, Guanghong Zhou, Zhi Li, Chuanguang Zhou.

**Project administration:** Tong Ren, Tianlin Guo, Yongqing Jiao.

**Formal analysis:** Guanghong Zhou, Chuanguang Zhou.

**Supervision:** Chunyan Hu, Junfeng Zhao.

**Validation:** Junfeng Zhao.

**Visualization:** Junfeng Zhao.

**Resources:** Tianlin Guo, Yongqing Jiao.

**Software:** Tianlin Guo.

**Data curation:** Xun Wang.

**Writing – original draft:** Chunyan Hu, Xun Wang.

**Writing – review & editing:** Chunyan Hu, Xun Wang.
